# In vitro activation of the CB_1_ receptor by the semi‐synthetic cannabinoids hexahydrocannabinol (HHC), hexahydrocannabinol acetate (HHC‐O) and hexahydrocannabiphorol (HHC‐P)

**DOI:** 10.1002/dta.3750

**Published:** 2024-06-19

**Authors:** Mattias Persson, Robert Kronstrand, Michael Evans‐Brown, Henrik Green

**Affiliations:** ^1^ Department of Forensic Genetics and Forensic Toxicology National Board of Forensic Medicine Linköping Sweden; ^2^ Division of Clinical Chemistry and Pharmacology, Department of Biomedical and Clinical Sciences, Faculty of Medicine and Health Sciences Linköping University Linköping Sweden; ^3^ European Monitoring Centre for Drugs and Drug Addiction (EMCDDA) Lisbon Portugal

**Keywords:** cannabinoid receptor agonists, CB_1_ receptor, designer drugs, illicit drugs, semi‐synthetic cannabinoids

## Abstract

Semi‐synthetic cannabinoids (SSCs) including hexahydrocannabinol (HHC) are emerging on the drug market and sold openly as purportedly legal replacements for cannabis and Δ^9^‐THC. By the beginning of 2024, 24 European countries had identified HHC, often sold openly in edibles (foods/candy), vapes and low‐THC cannabis flowers and resins. The SSC market is developing rapidly, with HHC acetate (HHC‐O), hexahydrocannabiphorol (HHC‐P) and others recently identified. These developments may mark the first major change in the market for ‘legal’ replacements to cannabis since ‘Spice’ containing synthetic cannabinoids, such as JWH‐018, emerged in 2008. Currently, there are some data available on the pharmacology of SSCs, which is crucial for understanding their effects, evaluating health risks and informing public health responses. This study focused on characterizing the in vitro activation of the human CB_1_ receptor by the (*R*)*‐* and (*S*)*‐*epimers of HHC, HHC‐P and HHC‐O. Using recombinant CHO‐K1 cells expressing the human CB_1_ receptor, the potency (EC_50_) and efficacy were determined. It was established that (9*R*)‐HHC and (9*R*)‐HHC‐P activated the CB_1_ receptor as partial agonists and with five and two times lower potency compared to JWH‐018, respectively, while the (*S*)*‐*epimers exhibited even lower potency. The (*R*)*‐*epimer of HHC‐O activate the CB_1_ receptor to even lesser extent and the (*S*)‐epimer showed no activation. For HHC and HHC‐P, all epimers exhibited similar level of efficacy. This available evidence suggests cannabimimetic effects of the tested SSC with the exception for the acetates that likely function as pro‐drugs in vivo.

## Graphical abstract

The semi‐synthetic cannabinoids market is developing rapidly, and 24 European countries have identified HHC, often sold openly. This study characterized the in vitro activation of the human CB_1_ receptor by the (9*R*) and (9*S*) epimers of HHC, HHC‐P and HHC‐O. HHC and HHC‐P activate the CB_1_ receptor as partial agonists suggesting cannabimimetic effects.

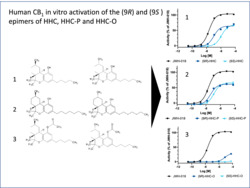

## 1 INTRODUCTION

**FIGURE 1:**
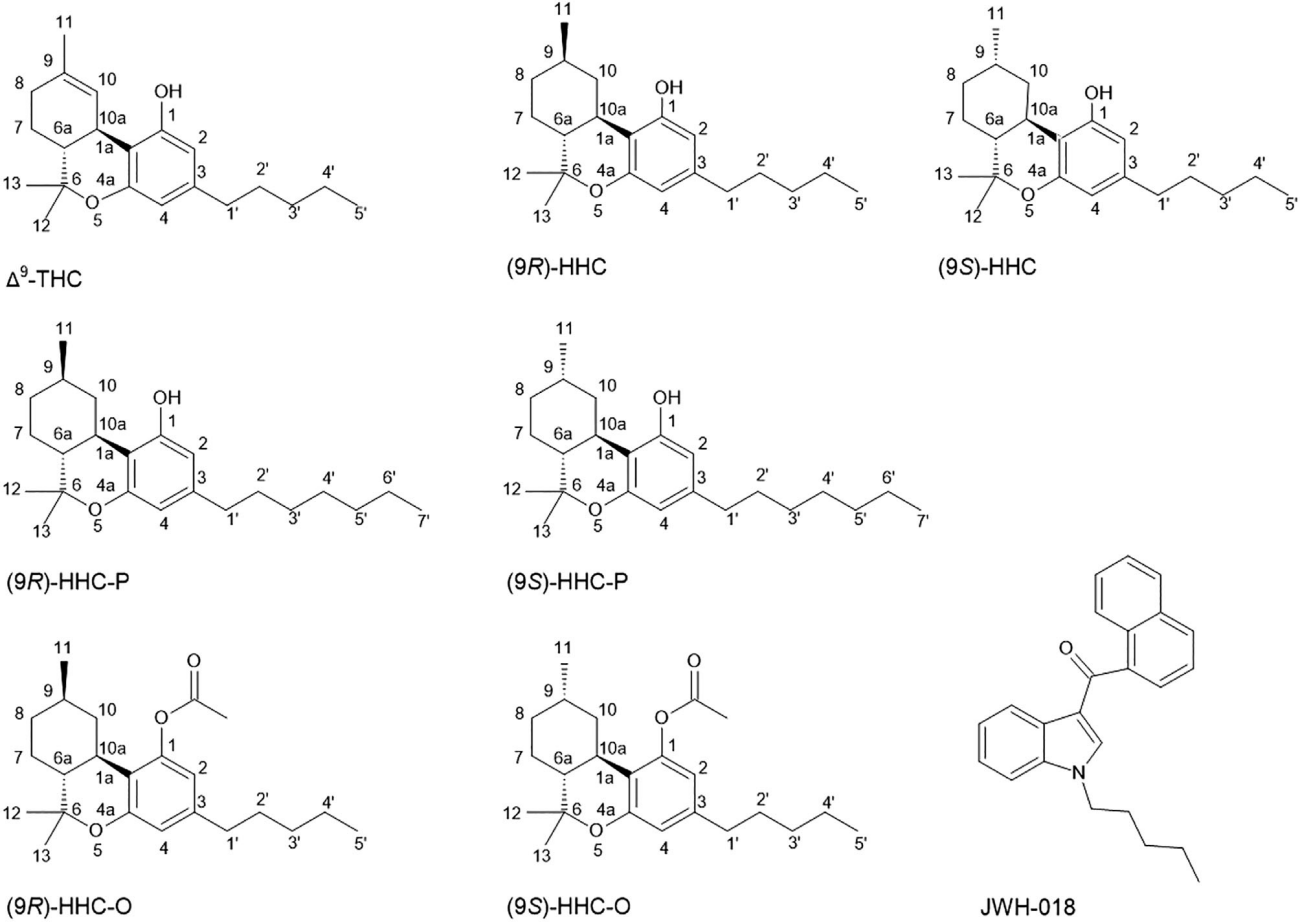
Chemical structures of Δ^9^‐THC, (9*R*)‐HHC, (9*S*)‐HHC, (9*R*)‐HHC‐P, (9*S*)‐HHC‐P, (9*R*)‐HHC‐O, (9*S*)‐HHC‐O and JWH‐018. [Correction added on 2 July 2024, after first online publication: The chemical structures of (9R)‐HHC and (9S)‐HHC in Figure 1 has been updated.]

In 2018, hemp legalization in the United States led to a series of semi‐synthetic cannabinoids (SSCs) emerging on the drug market and sold openly as legal replacements for cannabis containing Δ^9^‐THC (Figure 1).^1^, ^2^ The SSCs are mainly synthesized from cannabidiol (CBD) extracted from low Δ^9^‐THC hemp. Δ^8^‐THC emerged first in 2019 and hexahydrocannabinol (HHC) in 2021, and by February 2024, 24 European countries had also identified HHC in a range of products. The SSC market is still evolving rapidly, with a total of nine substances identified on the European drug market as of 30 April 2024: HHC, HHC acetate (HHC‐O), hexahydrocannabiphorol (HHC‐P), tetrahydrocannabidiol (H4‐CBD), Δ^9^‐tetrahydrocannabiphorol (Δ^9^‐THCP), hexahydrocannabihexol (HHCH), Δ^8^‐tetrahydrocannabiphorol (Δ^8^‐THCP), Δ^9^‐tetrahydrocannabutol (Δ^9^‐THCB) and 9‐hydroxyhexahydrocannabinol (9‐OH‐HHC) (European Monitoring Centre for Drugs and Drug Addiction (EMCDDA)). This study focused on HHC, HHC‐O and HHC‐P.

The synthesis and cannabimimetic properties of HHC was first described by Adams et al^3^ and Todd et al in 1940,^4^ and its chemistry and pharmacology has been reviewed by Ujváry.^5^ HHC has three stereogenic carbons, but in products currently available on the drug market, only the (9*R*)‐ and (9*S*)‐epimers with the 6a and 10a carbons both having the (*R*)‐configuration have been identified.^6^ Pharmacologically, HHC and HHC‐P are classified as cannabinoids. The activation of the CB_1_ and CB_2_ receptors has been studied in vitro showing that both (9*R*)‐HHC and (9*S*)‐HHC are partial agonists similar to Δ^9^‐THC but with lower potency, the (9*S*)‐epimer being the least potent.^7^ Radioligand binding affinity studies have also been performed for HHC^7^ but not for HHC‐P. HHC and Δ^9^‐THC also exhibit affinity for the μ opioid receptor.^8^ Similar to other cannabinoids, HHC and HHC‐P are non‐polar, lipophilic substances, and it is expected that they have low water solubility and high lipid solubility similar to Δ^9^‐THC and CBD.^5^, ^9^

HHC and other cannabinoid analogues have been explored in silico and in vitro studies as potential treatments for Alzheimer's disease due to their ability to inhibit acetylcholinesterase.^10^

The potential therapeutic use was also recently suggested by Durydivka et al. in their in vitro studies, they found that (9*R*)‐HHC exhibits similar activation of the CB_1_ receptor concerning the G‐protein signalling pathway but activates beta‐arrestin interactions more effectively than Δ^9^‐THC. They postulate that these differences may be important in developing treatments targeting the endocannabinoid system as they may have fewer side effects compared to existing cannabinoids.^11^

Nevertheless, the SSCs are all monitored as new psychoactive substances through the European Union (EU) Early Warning System on New Psychoactive Substances. This system allows Europe to rapidly detect, assess and respond to the health and social harms of new substances that appear on the drug market.^12^

SSC are sold openly as purportedly legal replacements for Δ^9^‐THC and cannabis in a range of highly attractive branded and unbranded products, some of which are sold as ‘legal highs’. These include foods (commonly known as edibles, such as gummies and marshmallows), vapes and concentrated oils, as well as hemp sprayed or mixed with SSCs (which looks and smells like cannabis). Products are sold in a range of brick‐and‐mortar and online shops, particularly those specialized in selling low‐THC cannabis and CBD products, as well as vaping products (‘smoke shops’) and vending machines.

The marketing of these products often makes direct comparison to the effects of Δ^9^‐THC and cannabis, but human dosing studies on the effects are lacking. However, in a recent report by Schirmer et al, one volunteer reported the effects after vaping 15 mg of HHC, containing 66% (9*R*)‐HHC, to be mildly cannabimimetic with a duration of about 2 h, while oral ingestion of 20 mg of HHC, containing 47% (9*R*)‐HHC, reportedly had no noticeable effects.^13^

From self‐reported data, the effects of HHC seem similar to those of cannabis with relaxation and euphoria the most frequently reported subjective effects. Even though non‐medical reasons for using HHC dominated, some users described medical reasons such as to treat anxiety, stress and pain.^14^ There are no studies on the effects of HHC‐P nor HHC‐O in humans.

Concerns exist that there could be a large demand for SSC products.^1^ This includes existing cannabis users and new consumers attracted to SSCs claimed effects and legal status—some of them young or otherwise inexperienced in drug use.^15^, ^16^, ^17^, ^18^ In some cases, ease of access to products, such as from high street CBD and vape shops as well as vending machines, may promote use.

These developments may mark the first major change in the market for ‘legal’ replacements to cannabis since ‘Spice’ products containing synthetic cannabinoids, such as JWH‐018, emerged 15 years ago in 2008.^19^ The synthetic cannabinoids, being highly potent and full agonists at the CB_1_ receptor, may cause more pronounced and severe acute toxicity than cannabis. Numerous reports of adverse effects, including severe poisonings and deaths, sometimes manifesting as outbreaks of mass poisonings, have been reported in the literature.^20^, ^21^, ^22^ Therefore, it is possible that SSCs may be seen by some consumers as safer alternatives.

However, emerging data have linked HHC and other SSCs with a range of adverse effects that appear to be typical of cannabimimetics. These range from mild and self‐limiting to cases of severe acute poisoning.^14^, ^16^

Ferretti et al, in a small online survey of self‐reported use patterns and subjective effects of HHC products in 109 individuals from the United States, found that approximately 17% of the respondents reported adverse effects, mostly sleepiness, red eyes, dry mouth and stomach problems. Interestingly, 22% of 54 individuals who reported HHC cessation also reported ‘withdrawal symptoms’. The most common symptoms were sleep difficulties and depressed mood.^14^

In addition, acute poisonings and psychotic episodes, sometimes requiring hospitalization, have been associated with HHC.^14^, ^15^, ^23^, ^24^ Poison centers in France and the Czech Republic have both reported an increase in poisonings to HHC since it emerged on the drug market. Although the data are limited, where known, many of the cases appear to be related to use of edibles (such as sweets/candies) and vapes containing HHC. In France, a case series of 37 poisonings found that 18 (49%) of cases were classed as moderate (16) or severe (2) on the poisoning severity score.^16^ [Correction added on 19 August 2024, after first online publication: In the preceding sentence, the information “19 (51%) of cases were classed as moderate (16) or severe (3)” has been corrected to “18 (49%) of cases were classed as moderate (16) or severe (2)”.] Separately, a case report related to a regular consumer of HHC products also noted severe effects including loss of consciousness, tonic–clonic crises, metabolic acidosis and rhabdomyolysis after vaping HHC, which was confirmed at 11 ng/ml plasma at admission to hospital.^25^ In some cases, poisonings have involved young people, including children.^14^, ^26^ For example, over a 20‐month period between June 2022 and the beginning of February 2024, the Czech poison centre recorded 170 cases of poisonings, mainly in children and adolescents.^26^[Correction added on 2 July 2024, after first online publication: The phrase ‘over an 8‐month period’ was corrected to ‘over a 20‐month period’ in the preceding sentence.]

Currently, there are limited data on the pharmacology of SSCs. Such data are essential for public health and policymakers in order to understand their effects, assess health risks and inform responses in a timely manner.^12^ This study characterized the in vitro activation of the human CB_1_ receptor by the (9*R*)‐ and (9*S*)‐epimers of HHC, HHC‐P and HHC‐O (Figure 1).

## 2 MATERIAL AND METHODS

Recombinant CHO‐K1 cells (AequoScreen®) expressing the human CB_1_ receptor (ES‐110‐AF) from Revvity (Waltham, Massachusetts, USA) were used to determine the potency (EC_50_) and efficacy compared to JWH‐018 (% activity) via luminescent analysis in three independent experiments. The method was carried out according to the manufacturer and have been described before.^27^ Each epimer was investigated in dilution series ranging from 29 pM to 60 μM in triplicates. To minimize the substances' ability to bind to plastics, pipette tips and 96‐well reaction plate were silanized with n‐hexane and dichlorodimethylsilane (99.5%) in 9:1 ratio. The dilution curves were performed in glass vials.

### 2.1 Cell culture media and chemicals

Cell culture media and assay media DMEM/Ham's F12 with 15 mM HEPES, L‐glutamine and without phenol red with and without 0.05% protease‐free BSA and fetal bovine serum (FBS) were from Thermo Fisher (Gothenburg, Sweden). Digitonin, trypsin and protease‐free bovine serum albumin (BSA) and the silinization chemicals n‐hexane and dichlorodimethylsilane were purchased from Sigma‐Aldrich (Darmstadt, Germany). Coelenterazine was from the Nanolight Tech (Pinetop, AZ, USA). Substances (9*R*)‐hexahydrocannabinol, (9*R*)‐hexahydrocannabiphorol, (9*S*)‐hexahydrocannabiphorol, (9*S*)‐hexahydrocannabiphorol, (9*R*)‐hexahydrocannabinol acetate and (9*S*)‐hexahydrocannabinol acetate were purchased from Cayman Chemical (Ann Arbor, Michigan, USA), (9*S*)‐hexahydrocannabinol and JWH‐018 from Chiron AS (Trondheim, Norway). The purity for all substances was between 98.2% and 100%.

### 2.2 Cell lines and dose–response assays

The cell lines were cultured in Ham's F12 media supplemented with 10% FBS at 37°C in a humidified air atmosphere containing 5% CO_2_. Coelenterazine (500 μM) were prepared in methanol and protected from light and 50 mM digitonin in DMSO, and both were stored at −20°C until use.

On the day of the dose–response assays, the cells were cultured to a confluency of 70%–90% and trypsinated, centrifuged at 200 *g* for 5 min at ambient temperature, resuspended at a concentration of 3 × 10^5^ cells/mL in pre‐warmed DMEM/Ham's F12 without phenol red supplemented with 15 mM HEPES, L‐glutamine and 0.05% protease‐free BSA (assay media). The cells were gently pre‐incubated with coelenterazine (2.5 μM) on a rotating wheel at room temperature at an angel of 10% for 3 h while protected from light. A dose–response curve with eight different concentrations were prepared in 96‐well‐plates (OptiPlate‐96 [LBS]), white opaque microplates from PerkinElmer, in triplicate, with a dilution of 1:8 between each well. JWH‐018 was analysed in the same concentration range to serve as CB_1_ receptor agonist reference. Wells containing cells and medium without drug were used as negative controls. As positive control, digitonin (67 μM) was used. Using a TECAN Spark 10 M (Tecan, Switzerland), the receptor activation at each drug concentration was determined by dispensing 50 μl of cells into each well (15,000 cells/well) and registering 200 luminescence readings for ~25 s. Baseline was set by measuring the luminescence for 10 cycles before the cells were added.

### 2.3 Data analysis

Area under the curve for the luminescence data from each well was determined. Response signals were normalized to the signal of three independent wells containing JWH‐018 at 60 μM and denoted as 100% activity. EC_50_ values and efficacy with 95% confidence intervals (profile likelihood) and curve fittings (non‐linear fit, three parameters) were calculated using all data points and the software GraphPad Prism version 10.2.0 for Windows (GraphPad Software, La Jolla, CA, USA). Differences in potency and efficacy between the compounds was evaluated using the log (EC_50_) values from the regression and compared to the potency of JWH‐018 using a Brown–Forsythe and Welch ANOVA with Dunnett T3 correction for multiple tests.

## 3 RESULTS AND DISCUSSION

**FIGURE 2:**
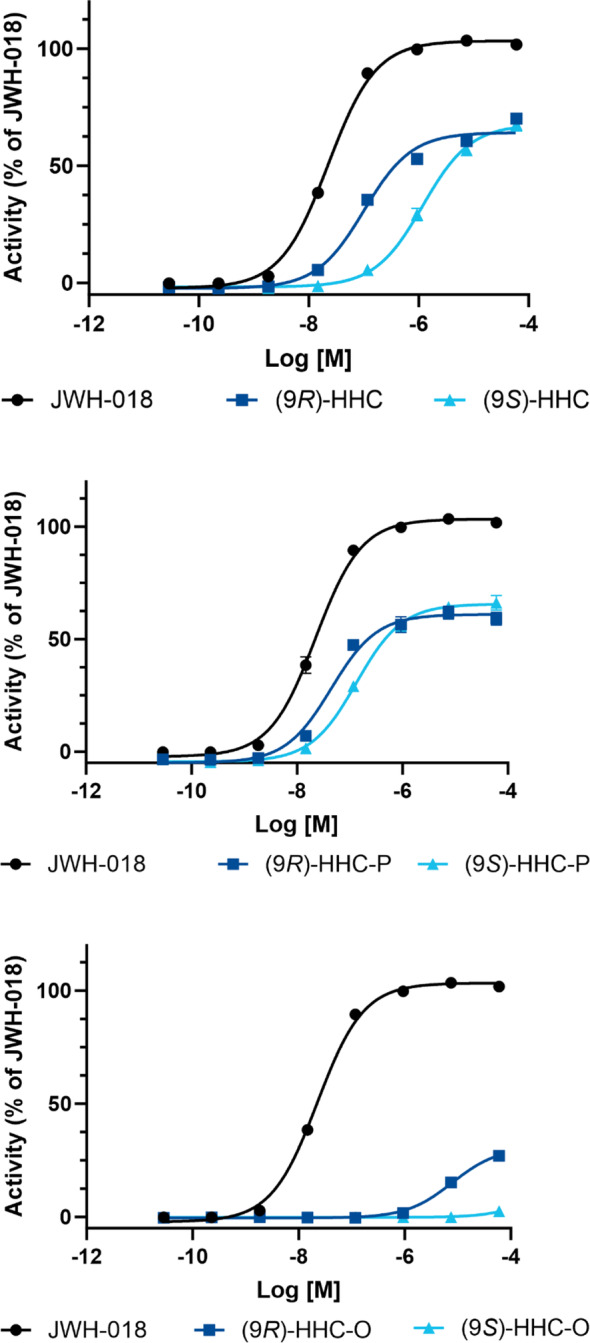
Pharmacological characterization of human CB_1_ receptor activation of the (9*R*)‐ and (9*S*)‐epimers of the semi‐synthetic cannabinoids (SSCs) included in this study. JWH‐018 is used as a reference.

In this study, the in vitro activation of human CB_1_ receptor was quantified using the individual epimers (9*S*)‐HHC, (9*R*)‐HHC, (9*S*)‐HHC‐P, (9*R*)‐HHC‐P, (9*S*)‐HHC‐O and (9*R*)‐HHC‐O. All the tested SSCs, except one, were found to be partial agonists compared to JWH‐018 (EC_50_ of 22.5 nM, 95% CI 19.9–25.6 nM) (see Figure 2 and Table 1). JWH‐018 was one of the first synthetic cannabinoids to emerge on the drug market around 2008 in Spice‐type ‘legal high’ products.^28^, ^29^ Subsequently, it has been used as a comparator substance in in vitro experimental studies of CB_1_ activity for other cannabinoids that emerged.^27^, ^28^, ^29^, ^30^ In a comparison between JWH‐018 and Δ^9^‐THC, it was concluded that JWH‐018 has an in vivo ED_50_ that is about two times lower in mouse than Δ^9^THC and about four times higher binding affinity than Δ^9^THC.^31^

**TABLE 1:** Pharmacological characterization of human CB_1_ receptor activation of the semi‐synthetic cannabinoids (SSCs) included in this study. The difference in potency (EC_50_) and efficacy, together with the *P*‐values for the statistics are compared to the effects of JWH‐018 as a reference.

Substance	EC_50_ (95% CI) nM	EC_50_ *P*‐value	Efficacy (95% CI) %	Efficacy *P*‐value
(9*R*)‐HHC	101 (81.9–125)	<0.0001	64 (62–66)	<0.0001
(9*S*)‐HHC	1190 (951–1490)	<0.0001	68 (65–71)	<0.0001
(9*R*)‐HHC‐P	44.4 (33.6–58.7)	0.0063	61 (58–64)	<0.0001
(9*S*)‐HHC‐P	134 (109–164)	<0.0001	66 (63–68)	<0.0001
(9*R*)‐HHC‐O	>33,000	—	>27	—
(9*S*)‐HHC‐O	n.d.	—	n.d.	—
JWH‐018	22.5 (19.9–25.6)		103 (102–105)	

Abbreviation: n.d., not determined.

The highest potency of all the tested substances was identified for (9*R*)‐HHC‐P that exhibited a potency with EC_50_ of 44.4 nM (33.6–58.7 nM 95% CI) and was higher than the corresponding (9*S*)‐HHC‐P (EC_50_ 134 nM, 95% CI 139–164 nM). A similar effect was found for HHC, where (9*R*)‐HHC exhibited an EC_50_ at 101 nM (95% CI 81.9–125 nM), and (9*S*)‐HHC exhibited an EC_50_ of 1190 nM (95% CI 951–1,490 nM). (9*S*)‐HHC‐O did not activate the CB_1_ receptor, whereas a minor activation to an efficacy of at least 27% was identified for (9*R*)‐HHC‐O, with a calculated EC_50_ without a clear plateau of >33,000 nM. A potential explanation of the activity of (9*R*)‐HHC‐O, since it is a prodrug, could be the presence of (9*R*)‐HHC as an impurity; however, an analysis of (9*R*)‐HHC‐O in cell media using LC‐MS did not show any indication of hydrolysis (data not shown). Although there were major differences between the EC_50_ values, no significant difference in efficacy between the HHC and HHC‐P epimers was detected and varied from 61% (95% CI 58%–64%) for (9*R*)‐HHC‐P to 68% (95% CI 65%–71%) for (9*S*)‐HHC (Table 1). However, compared to JWH‐018, efficacies were all 34%–39% lower. At high doses, all HHC and HHC‐P epimers had about the same maximal activation, indicating that the dosage might have a high impact for the effect. During the metabolism of the HHC acetates, it has been shown that HHC was formed rapidly in human hepatocytes, suggesting that this might apply to humans following ingestion.^32^

Similar to THC, HHC and related substances are non‐polar^10^ and may adhere to plastics used in experiments. Therefore, the experimental procedure was improved with the use of glass vials and silanization of plastic. Without this approach, the adsorption of the substances to surfaces resulted in erroneous low efficacy (37%) and potency (EC_50_ 144 nM for (9*R*)‐HHC) compared to the improved procedure with a 64% efficacy and an EC_50_ 101 nM. Therefore, minimizing the exposure to plastics and silanization are recommended when studying non‐polar substances.

For each compound, the (*R*)‐form exhibited a lower EC_50_ value. The higher potency of (9*R*)‐HHC compared to (9*S*)‐HHC is in accord with earlier studies of SSCs.^6^, ^7^ The study of Russo et al used behavioral tests on mice for in vivo determination of the cannabinoid profles.^11^ In the study of Nasrallah and Garg,^12^ it was determined that Δ^9^‐THC and (9*R*)‐HHC exhibited about the same potency and that the (9*S*)‐HHC was about 16‐fold lower by the use of a G‐protein coupled receptor (GPCR) functional assay. Also, in their experiments for CB_1_ activity, the efficacy was about the same for both HHC epimers and for Δ^9^‐THC.^12^ During the revision of the current paper, two studies that used the beta‐arrestin pathway were published: Durydivka et al^11^ and Janssens et al^33^ Our results are in accord with those from Janssens et al that (9*R*)‐HHC and (9*R*)‐HHC‐P are both more potent than their corresponding (*S*)‐epimers and that (9*R*)‐HHC‐P was more potent than (9*R*)‐HHC. However, in this assay (using the G‐protein pathway), (9*S*)‐HHC‐P was also more potent than (9*S*)‐HHC, whereas in the beta‐arrestin assay, they presented with equal EC_50_ values.^33^ In the study by Durydivka et al, the potency order using both the G‐protein activation and beta‐arrestin activation pathways was the same as in this data set with (9*R*)‐HHC being more potent than (9*S*)‐HHC.^11^ These differences in activation pathways may be important when discussing adverse effects and potential harm and add to the general fact that the epimers have different potencies at CB_1_ and may be present in different ratios in products.

Nasrallah and Garg^7^ identified a ratio ranging from 0.2:1 to 2.4:1 of (9*R*)‐HHC to (9*S*)‐HHC when investigating the content in a number of commercially available HHC products, indicating that products can have varying and unpredictable potency. Seized material labelled as HHC in Belgium had a ratio of (*S*) to (*R*) epimers with a ratio ranging from 0.27 to 0.89.^33^ [Correction added on 2 July 2024, after first online publication: The phrases ‘theoretical’ and ‘theoretical ratios of’ were removed and the ratios have been changed to ‘0.27 to 0.89’ in the preceding sentence.] This poses an inherent risk of poisoning for consumers, as they will be unaware of the differences in strength between products.^34^

In vitro and in vivo animal laboratory studies suggest that HHC has broadly similar pharmacological effects to Δ^9^‐THC.^5^ In a mouse model using a racemic mixture of (9*S*)/(9*R*)‐HHC, it was found that HHC was less potent than THC in the tetrad test and that it did not induce antinociception.^35^ In another study by Russo, the (9*R*)‐epimer was active in all parts of the tetrad test, including antinociception.^6^ Although the pharmacological and behavioral effects of HHC in humans have not been studied, recent anecdotal self‐reports indicate that its effects are similar to that of cannabis and Δ^9^‐THC.^14^ This also appears to be consistent with the clinical features from acute poisonings reported in the literature.^5^ However, further data are required to allow a systematic and detailed comparison with cannabis and Δ^9^‐THC. As polysubstance use is increasingly the norm in society and that HHC and other SSC products may be adulterated or contaminated with other cannabimimetic substances, including Δ^9‐^THC and Δ^8^‐THC, as well as other novel SSCs,^16^ studies should include the comprehensive analysis of both products and biological samples in order to ascertain which substances and doses consumers have been exposed to.

## 4 CONCLUSION

Following a minor change in the experimental protocol to avoid adherence to plastics used in experiments, it is concluded that HHC and HHC‐P activate the CB_1_ receptor as partial agonists and with lower potency compared to JWH‐018. The available evidence suggests cannabimimetic effects of the tested SSC, with the exception of HHC‐O. There was a difference in the potency of (*S*)‐ and (*R*)‐epimers, where (9*R*)‐epimers were more potent. (9*S*)‐HHC‐O did not activate the CB_1_ receptor. The HHC acetates are likely to act as pro‐drugs and are likely converted into HHC in vivo. This study confirms the information on the pharmacological mechanisms of action of HHC and HHC‐P and showed that HHC‐O activation of the CB_1_ receptor was little or absent. Such data are essential for the interpretation of toxicological findings, as well as supporting early warning and risk assessment of the health and social risks.

## CONFLICT OF INTEREST STATEMENT

Nothing to disclose.
